# Potential of a gypsum-free composting process of wheat straw for mushroom production

**DOI:** 10.1371/journal.pone.0185901

**Published:** 2017-10-05

**Authors:** Thibaut M. B. Mouthier, Baris Kilic, Pieter Vervoort, Harry Gruppen, Mirjam A. Kabel

**Affiliations:** 1 Wageningen University and Research, Laboratory of Food Chemistry, Wageningen, The Netherlands; 2 CNC Grondstoffen B.V., Milsbeek, The Netherlands; Tallinn University of Technology, ESTONIA

## Abstract

Wheat straw based composting generates a selective substrate for mushroom production. The first phase of this process requires 5 days, and a reduction in time is wished. Here, we aim at understanding the effect of gypsum on the duration of the first phase and the mechanism behind it. Hereto, the regular process with gypsum addition and the same process without gypsum were studied during a 5-day period. The compost quality was evaluated based on compost lignin composition analysed by py-GC/MS and its degradability by a commercial (hemi-)cellulolytic enzyme cocktail. The composting phase lead to the decrease of the pyrolysis products 4-vinylphenol and 4-vinylguaiacol that can be associated with *p*-coumarates and ferulates linking xylan and lignin. In the regular compost, the enzymatic conversion reached 32 and 39% for cellulose, and 23 and 32% for xylan after 3 and 5 days, respectively. In absence of gypsum similar values were reached after 2 and 4 days, respectively. Thus, our data show that in absence of gypsum the desired compost quality was reached 20% earlier compared to the control process.

## Introduction

The production of substrate used for the growth of the mushroom *Agaricus bisporus* is based on a composting process, which involves the bioconversion of a mixture of wheat straw with horse and chicken manure, water and gypsum [1]. The first phase of this biological process requires 5 days before the compost is considered ready for the second phase, and a time-reduction of the first phase would be desirable. In our research, we aim at understanding the effect of gypsum on the duration of the first phase, and plant cell wall structure modifications required for an optimal compost production.

Wheat straw is the second largest biomass feedstock in the world [2] and is a byproduct of the agricultural industry. Nowadays, amongst others, it is used for animals as feed or bedding. Wheat straw is a lignocellulosic feedstock and mainly composed of cellulose (33–40%w/w), hemicellulosic xylan (20–25%w/w) and lignin 15–20%w/w) as reviewed by Prasad and Singh et al. (2007) [3]. Wheat straw is one of the main ingredients of the composting process studied and serves as main carbon source for a wide range microorganisms and the mushroom *A*. *bisporus*. In addition to wheat straw, gypsum and water, also horse and chicken manure are added. Additionally, part of the wheat straw has already been mixed with horse manure originating from horse bedding and is considered as “not fresh”. The manure supplements the compost with microorganisms and ammonium [4] and is the main nitrogen source. Gypsum provides a calcium source for mushrooms and, in addition, is known to lower the pH [1,4].

After mixing all ingredients, the composting process follows three phases [5]. The first phase is a thermo-biological treatment (Phase I), the second phase is used to condition the compost and to enable microorganisms other than *A*. *bisporus* to grow (Phase II). It is conducted for 5 to 9 days where the temperature is maintained at 56°C for 24 h before cooling to room temperature by blowing indoor air.During the third phase (Phase III),mycelium growth of *A*. *bisporus* within the compost is achieved (Phase III). The mycelium spread within the compost and fully colonize it after 12 to 16 days. The latter two phases (Phase II and III) have been described previously. In particular the fate of compost content and composition over the three phases have already been studied [5] and will not be further discussed here. At industrial scale, Phase I is conducted in 180 tons compost capacity tunnels for 3 to 6 days in presence of oxygen. This thermo-biological phase aims to soften the wheat straw structure for the following phases through the action of ammonia [1]. Within the first 24 hours of phase I, microorganisms from the manure will grow releasing ammonia and generating heat up to 80°C, consequently softening the mixture. As mesophilic microorganisms will grow from the initial starting temperature, the increase of temperature lead to their replacement by thermophilic microorganisms such as *Aspergillus fumigatus* and *Myceliophtora thermophila* [1,6,7]. The growth of microorganisms within the compost during phase I will enhance the degradation of the carbohydrate polymers and the decomposition of organic nitrogen releasing gaseous ammonia through ammonification and immobilisation increasing temperature and pH [1,4,8].

In the field of thermo-chemical conversion of lignocellulosic feedstocks into fermentable carbohydrates, meant for the production of biofuels or biochemicals, pretreatments with ammonia resulting in a pH above 8 were shown to increase enzymatic hydrolysis of (hemi)cellulose by (partial) delignification [9,10,11]. In these studies, pretreatment and enzymatic conversion aimed at a complete deconstruction of carbohydrates into monosaccharides and, therefore, required a more than five times higher ammonia loading than achieved in the Phase I composting process. Whether lignin structures become modified in compost during Phase I to improve carbohydrate accessibility is unknown. However, the latter has been studied during mushroom growth, such as for *A*. *bisporus* [12] and *Pleurotus eryngii* [13]. In these studies pyrolysis GC-MS was used to monitor lignin modifications.

In our research, it is hypothesized that higher ammonia loadings in Phase I would help to open up the structure, possibly by lignin modifications, to enable the development of the microorganisms in Phase II. In addition, it is hypothesized that a higher amount of ammonia is reached when pH is not controlled by gypsum. The amount of ammonia needed in order to achieve Phase I compost quality (5 composting days) is unknown.

The objective of this research is to assess the potential of biological ammonia pretreatment (Phase I) of a wheat straw based composting process by a gypsum free Phase I process. The gypsum free process was compared to the regular (control) process, in which gypsum was added. Lignin was fingerprinted with analytical pyrolysis GC-MS. The quality of the compost obtained at various time points during Phase I was evaluated based on its enzymatic degradation by using a commercial (hemi-)cellulolytic cocktail.

## Material and methods

### Sample of Phase I compost process in presence and absence of gypsum

Wheat straw and compost samples were obtained from CNC (CNC Grondstoffen B.V., Milsbeek, The Netherlands) in 2013 and in 2015. CNC is a worldwide supplier of substrate for mushroom growing.

The composting process of Phase I at CNC was conducted according to the same methodology as described by Jurak et al. (2014) [5]. Both in 2013 and 2015, composting tunnels were conducted in parallel. The ingredients, wheat straw, horse manure, chicken manure and water, were mixed and divided in two batches. One batch was mixed with gypsum and used to fill one tunnel (4 x 35 x 4.5 m). Samples taken from this tunnel were coded P-13 and P-15 for samples obtained in 2013 and 2015, respectively. The second batch was used to fill the second tunnel (absence of gypsum) and samples were coded as A-13 and A-15, respectively, for the 2013 and 2015 experiment. At the beginning of Phase I, the substrate of both tunnels had a 1:3 (w/w) solid-to-liquid ratio. The circulation of air inside both tunnels was performed in a closed cycle to avoid ammonia losses.

The temperature of the compost was measured 50 cm below the surface in hexaplicate using a thermometer (Testo 110, Testo Inc., Sparta Township, NJ, United States). Ammonium and pH were measured according to Kjeldahl (1883) [14]. The ammonia in the air was measured using a Dräger tube (CH31901) (Drägerwerk AG & Co. KGaA, Lübeck, Germany) in the closed-recycling air system from each tunnel.

In the 2013 experiment, Phase I samples were collected every day from both tunnels and were coded as follows: start of Phase I (P0-13 and A0-13), 1 day (P1-13 and A1-13), 2 days (P2-13 and A2-13), 3 days (P3-13 and A3-13), 4 days (P4-13 and A4-13), and 5 days (P5-13 and A5-13). Samples from tunnel P-15 and A-15 were collected at the beginning and at the end of Phase I (P0-15 or A0-15 and P5-15 or A5-15 for the tunnel in presence of gypsum and absence of gypsum, respectively). For every sample, 3 times 10 kg were collected from 50 cm below the surface (within the first 5 meters of the tunnels due to high temperature) and mixed thoroughly. The samples were subdivided to 1 kg amounts and frozen immediately. Samples were freeze dried and milled (<1 mm) (Retsch Mill MM 2000, Retsch, Haan, Germany).

### Water extraction

Freeze dried and milled samples (10 g) were suspended in Millipore water (100 mL) overnight and were extracted at room temperature under continuous stirring. After centrifugation (10 000 x *g*; 15 min; 20°C), the residues were washed three times with 100 mL Millipore water. The residues were recovered as water un-extractable solids (WUS) after freeze dried.

Freeze dried WUS-samples were coded as follow: samples from 2013, start of Phase I (wP0-13 and wA0-13), 1 day (wP1-13 and wA1-13), 2 days (wP2-13 and wA2-13), 3 days (wP3-13 and wA3-13), 4 days (wP4-13 and wA4-13), and 5 days (wP5-13 and wA5-13). Samples from 2015, start of Phase I (wP0-15 and wA0-15) and 5 days (wP5-15 and wA5-15).

### Enzyme hydrolysis

WUS samples (25 mg) were suspended in 1 mL 50 mM sodium acetate buffer (pH 5.0). Samples were boiled for 10 min to stop remaining enzyme activity and to avoid growth of remaining microorganisms. Samples were then cooled to room temperature. Incubations were started by the addition of 2.7% w/w CellicCtec2 ((protein / dry matter (dm); CellicCtec2 protein concentration 127 mg mL^-1^) (Novozymes, Bagsværd, Denmark)) and 0.3% w/w CellicHTec ((protein / dm); CellicHTec protein concentration 120 mg mL^-1^) (Novozymes, Bagsværd, Denmark)) to the boiled and cooled sample. Samples were incubated for 24 h at 50°C in a head over tail rotator. Enzymes were inactivated (10 min at 100°C) prior to centrifugation (10 000 x *g*, 5 min). The supernatants were collected and subjected to further analysis. Enzyme treatment was performed in duplicate and replicated for the beginning and end of Phase I.

For xylanase treatment, WUS samples (15 mg) were suspended in 1.5 mL mM sodium acetate buffer (pH 5.0) were incubated for 24 hours at 50°C with a pure endo-(1,4)-β-d-xylanase 1 (EX1) (23 μL / 5 mg freeze dried WUS) from *Aspergillus awamori* (EC 3.2.18) (protein concentration 21.5 μg mL^-1^) [15]. Prior to enzyme addition, samples were put at 100°C for 10 min to inactivate remaining enzyme activity and cooled at room temperature. Samples were incubated for 24 h at 50°C in a head over tail rotator. Enzymes were inactivated (10 min at 100°C) prior to centrifugation (10 000 x *g*, 5 min). The supernatants were collected and subjected to further analysis. Treatment were performed in duplicates.

### Analytical methods

#### Neutral sugar content and composition

The neutral sugar content and composition was determined in duplicate according to Englyst and Cummings (1984) [16], using inositol as an internal standard. Samples were treated with 72% (w/w) H_2_SO_4_ (1 h, 30°C) followed by hydrolysis with 1 M H_2_SO_4_ for 3 h at 100°C, uronic acids released were analysed (section below) and the constituent sugars released were analysed as their alditol acetates using gas chromatography (ThermoScientific, Waltham, MA, USA). Total carbohydrate content was calculated as the sum of neutral carbohydrates and uronic acids.

#### Uronic acid content

Uronic acid content was determined in duplicate as anhydro-uronic acid content by an automated m-hydroxydiphenyl assay [17] with addition of sodium tetraborate using an autoanalyser (Skalar Analytical BV, Breda, The Netherlands). Glucuronic acid (Fluka AG, Busch, Switzerland) was used as a reference (0–100 μg mL^-1^).

#### Nitrogen and protein content

Samples were analysed in duplicate for nitrogen content in duplicate using the combustion (DUMAS) method on a Flash EA 1112 Nitrogen Analyzer (Thermo Scientific, Sunnyvale, CA, USA). Methionine (AcrosOrganics, Geel, Belgium) was used as a standard. Nitrogen to protein conversion factor of 6.25 was used (Jones, 1931) [18].

#### Insoluble (Klason) lignin content

To each sample of 300 mg dm, 3 mL of 72% w/w H_2_SO_4_ was added and samples were pre-hydrolysed for 1 h at 30°C. Distilled water (37 mL) was added to each sample and samples were put in a boiling water bath for 3 h and shaken every half hour. Next, the suspension was filtered over G4 glass filters (Duran Group, Wertheim/Main, Germany). The residual part was washed until it was free of acid and dried overnight at 105°C. Final residues were corrected for ash content and considered as a measure for the acid insoluble lignin content. Analysis was performed in duplicates.

#### Ash content

Freeze dried samples or lignin residues were first dried overnight at 105°C in the oven and weighed and, subsequently, stored at 575°C for 4 h and again weighed. The difference between the mass measured after incubation at 105°C and after incubation at 575°C was taken as ash content. Analysis was performed in duplicates.

#### High performance anion exchange chromatography

High performance anion exchange chromatography (HPAEC) was performed on a Dionex ICS-5000 unit (Dionex, Sunnyvale, CA, USA) equipped with a CarboPac PA-1 column (2 mm x 250 mm ID) in combination with CarboPac guard column and PAD detection (Dionex). The system was controlled by Chromelion software (Thermo Scientific, Sunnyvale, CA, USA). Elution and quantification of mono- and oligosaccharides was performed at 0.3 mL min^-1^ with a combination of 3 eluents, A: 0.1M NaOH; B: 1M NaOAc in 0.1M NaOH; C: H_2_O. The elution profile for the monosaccharides was as following: 0–30 min 15% A and 85% C, 30–35 min 100% B, 35–45 min 100% A, 45–60 min 15% A and 85% C. The elution profile for the oligosaccharides was as following: 0–35 min: 0–38% B mixed in A, 35–38 min 100% B, 38–50 min 100% A. For quantification, glucose, xylose, xylo-oligosaccharides (XOS) with a degree of polymerization (DP) of 2 to 4 (Megazyme, Wicklow, Ireland) and glucuronic acid were used for calibration in at least 4 increasing concentrations between 5 and 30 μg mL^-1^. Analysis was performed in duplicate.

#### Analytical pyrolysis GC/MS analysis

Pyrolysis of WUS-samples (80–100 μg) were performed in triplicates, weighed on a Mettler-Toledo XP6 microbalance (Mettler-Toledo, Columbus, US) was performed with an EGA/PY-3030D micro-furnace pyrolyzer (Frontier Laboratories, Fukushima, Japan) mounted on to a Thermo7820A gas chromatograph equipped with a DB-1701 fused-silica capillary column (60 m × 0.25 mm internal diameter, 0.25 μm film thickness) and coupled to a DSQ-II thermo mass selective detector (EI at 70 eV) (Thermo Scientific, Waltham, MA, USA). The pyrolysis was performed at 500°C. The GC oven temperature was programmed as follows: 70°C (2 min hold) linearly increased to 230°C at 5°C per min, and to 270°C at 2.5°C per min. Helium was the carrier gas (1 mL min^-1^). The compounds were identified by comparing their mass spectra with those of the Wiley and NIST libraries and with those reported in literature [19]. The relative area per compound identified was calculated according to Jurak et al. (2015) [12]. For all compounds identified spectra were checked manually.

## Results and discussion

### Composting conditions in Phase I in presence or absence of gypsum

**Fig 1:**
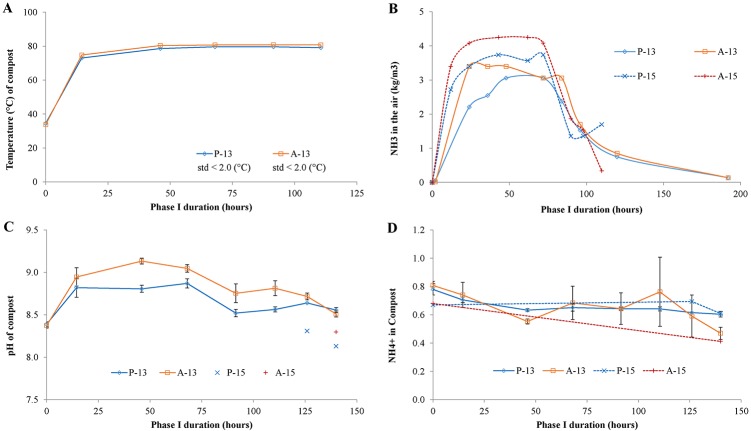
Conditions measured during the first 5-days of the composting process (Phase I) from a wheat straw-manure based mixture in presence (P) and in absence (A) of gypsum. A: Temperature (°C); B: Gaseous ammonia (NH_3_) measured in the air (kg/m^3^); C: pH of the compost; D: NH_4_^+^ content (%) in the compost, based on dry matter.

The composting process starts after mixing the ingredients (wheat straw, horse and chicken manure, water) and filling the Phase I-tunnel. In our research we compared a tunnel filled with mixed ingredients in absence of gypsum with a control tunnel filled with mixed ingredients and gypsum during Phase I. The hypothesis was that the absence of gypsum will lead to higher amounts of gaseous ammonia, higher compost pH, and will help opening up the compost structure enabling growth of microorganisms in Phase II, compared to the control tunnel. Hence, the conditions reached in both tunnels, including pH and ammonia content, were measured and the data are presented in Fig 1. From Fig 1A, it can be seen that in both tunnels (P-13 and A-13) the temperature increased within 24 h to 80°C and remained at that temperature till the end of Phase I. Such a temperature-profile is as expected for this phase and follows the observed pattern year round (CNC, The Netherlands, pers. comm.). The moisture content of compost from both tunnels (P-13 and A-13) was the same and remained constant during Phase I (73–75 w/w % (standard deviation < 0.1, S1 Table).

Gaseous ammonia (Fig 1B) and pH (Fig 1C) showed an increase for both tunnel (P-13 and A-13) within the first 24 h. In both tunnels, a similar trend was observed, but in the A-13 higher values for both pH and gaseous ammonia (pH 9 and 3.4 kg m^-3^) were reached after 24 hours Phase I compared to the P-13 tunnel (pH 8.8 and 2.2 kg m^-3^). Data from P-15 and A-15 confirmed the described trends of P-13 and A-13, showing a higher pH and higher gaseous ammonia in absence of gypsum. Previously, Gerrits (1988) [1] also showed that in absence of gypsum, pH and gaseous NH_3_ was higher compared to composting in presence of gypsum. The measurements of NH_4_^+^ in compost (Fig 1D) did not show any major differences between P-13 and A-13 compost (ranging from 0.8% to 0.6% NH_4_^+^) although the A-13 samples gave a relatively high standard deviation during Phase I compared to the P-13 samples. Nevertheless, comparing the end of A-13 and A-15 to the end of P-13 and P-15 (0.4–0.5% to 0.6% NH_4_^+^, respectively), it can be suggested that the absence of gypsum led to a slightly lower NH_4_^+^ in the compost. Such a lower NH_4_^+^ content matched well with the measured higher pH and gaseous ammonia in absence of gypsum because the dissociation of NH_4_^+^ in NH_3_ and H^+^ is favoured at high pH.

To summarize, the absence of gypsum in Phase I led to higher pH values and NH_3_ gas concentration compared to composting with gypsum, confirming our hypothesis, and thus affected the “chemical” severity of the process. The other treatment conditions such as temperature (°C) and moisture content (w/w %) were found to be similar and followed the same trend during Phase I, regardless the presence or absence of gypsum.

### Enzymatic hydrolysis of Phase I-compost obtained in presence or absence of gypsum

**Fig 2:**
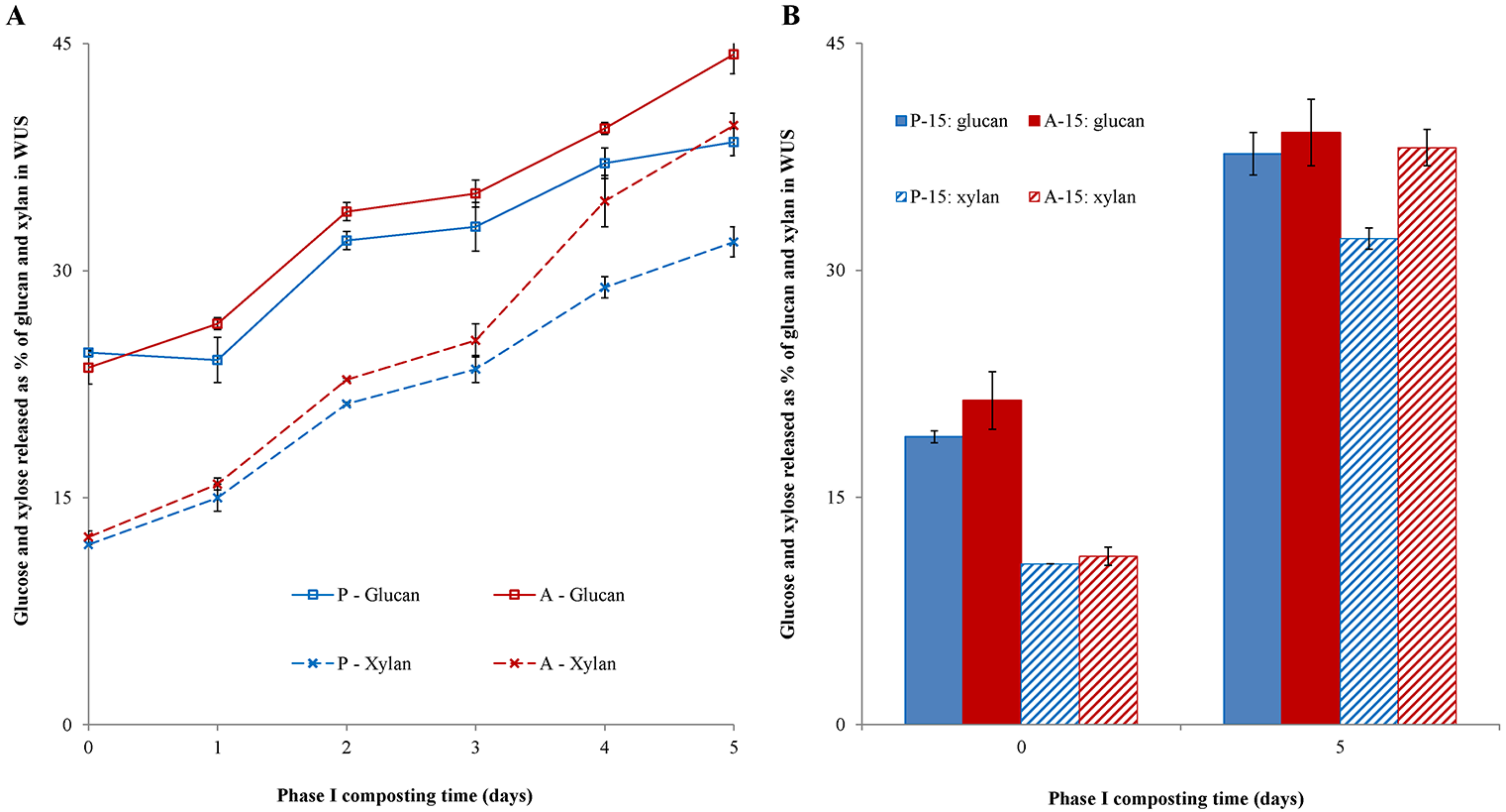
Enzymatic hydrolysis (CellicCTec2/ CellicHTec; 24 h) of WUS samples prepared from Phase I-compost expressed as glucose and xylose released (%) from total glucan and xylan in the corresponding WUS. Samples obtained from tunnels in presence (blue; wP-13 and wP-15) and in absence (red; wA-13 and wA-15) of gypsum. A: Phase I from 2013; B: Phase I from 2015.

As indicated in the previous section, the Phase I-samples obtained from the gypsum-free process were exposed to a higher pH and gaseous ammonia content compared to the Phase I-samples in presence of gypsum. To corroborate our main hypothesis that ammonia in Phase I helps to open up the structure to enable growth of the microorganisms in Phase II, the samples (either exposed to gypsum or not) were subjected to enzyme hydrolysis mimicking the microbial growth in Phase II. Only the enzymatic degradability of water unsoluble solids (WUS) was studied, because water soluble material is assumed to be well accessible for enzymes and microbes. The glucan to glucose and xylan to xylose conversions of enzymatic treated WUS are shown in Fig 2.

At the beginning of Phase I, as expected glucan (24.0% ± 0.6) and xylan (12.2% ± 0.3) conversions were similar for wP0-13 and wA0-13 (Fig 2). At the end of Phase I, glucan and xylan conversions were increased for wP5-13 (38.5% (± 0.9) and 31.9% (± 1.0), respectively). For wA5-13, conversions were even higher (44.3% (± 1.3) and 39.6% (± 0.8), respectively). The experiment and enzymatic hydrolysis was repeated in 2015. Glucan and xylan conversions of wP0-15 (19.0% ± 0.4 and 10.6% ± 0.0, respectively) were again similar to conversions of wA0-15 (21.4% ± 1.9 and 11.1% ± 0.6, respectively). At the end of Phase I, glucan and xylan conversions were found again to be higher for wP5-15 (37.4% ± 1.4 and 32.1% ± 0.7, respectively), and again even higher for wA5-15 (39.1% ± 2.2 and 38.1% ± 1.2, respectively).

Hence, it was concluded that compost of both tunnels (P-13 and A-13) became more enzyme degradable during Phase I, seen from the enzymatic glucan and xylan conversions of WUS (Fig 2) for both experiment performed in 2013 and 2015. Moreover, samples from the Phase I-tunnel in absence of gypsum resulted in WUS having higher glucan and xylan conversion compared to the WUS samples obtained in presence of gypsum, confirming our hypothesis.

Slight conversion differences for wP-15 and wA15 were observed compared to wP-13 and wA-13. These differences may be due to the initial difference in compost composition originating from straw, which is known to vary over the years [20,21]. Carbohydrate composition will be discussed and shown in a lower section.

Usually, Phase I compost in presence of gypsum is known (CNC, pers. comm.) to be ready for Phase II after 5 days, although also a 3-day-Phase I is occasionally applied. Now, it was assumed that glucan and xylan conversion of compost-WUS obtained after 3 or 5 days mimics the microbial growth in Phase II or the ‘readiness’ of Phase-I compost. The same conversions of WUS obtained from Phase-I in presence of gypsum (32.0 and 38.5% for glucan, and 23.0 and 31.9% for xylan conversion (wP3-13 and wP5-13, respectively)) were also reached one day earlier in absence of gypsum (33.9 and 39.4 for glucan and 22.8 and 34.6 for xylan conversion, wA2-13 and wA4-13 respectively). This higher enzyme degradability of the composts obtained in the gypsum-free Phase I compared to those from the gypsum-rich Phase I, can be the result of higher pH (pH >8) and higher gaseous ammonia in the gypsum-free process. The latter conditions, in particular the higher pH, have been shown to induce both de-acetylation of xylan and hydrolysis of the carboxyl-ester linkages present between arabinosyl-substituents of xylan and ferulate or *p*-coumarate and, possibly, between xylan and lignin [22,23,24]. Therefore, it can be contemplated that the absence or a reduced amount of ester-linkages present between xylan and lignin resulted in a more open structure of the lignocellulosic network of the compost and a higher accessibility of xylan for enzymes, which favoured glucan and xylan conversions. Additionally, ammonia can be expected to partly degrade the lignin present, again allowing a better accessibility for enzymes, which has been reported frequently in previous research for lignocellulosic feedstocks such as corn stover and *Miscanthus* [23,25]. It should be noted, however, that in the latter studies, aqueous ammonia is used at much higher concentration than reached in the current study. Hence, in our composting process, we expected a start of lignin deconstruction, which is discussed in more details in a lower section. Lignin deconstruction or partly degradation could lead to an increase in accessibility of xylans being linked to lignin via ferulates and *p*-coumarates [24].

**Fig 3:**
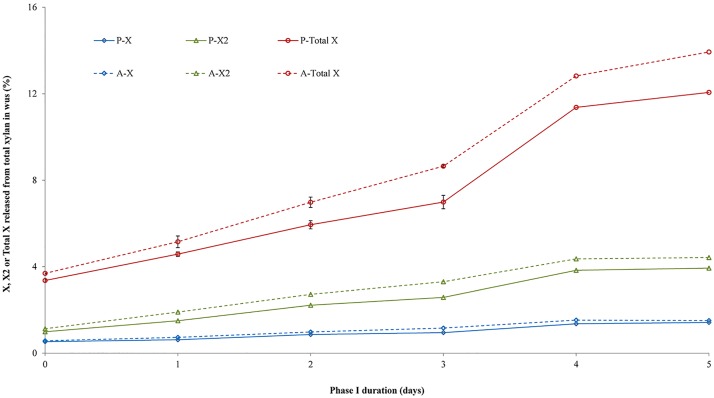
Endo-xylanase (*Aa*GH10) hydrolysis of WUS expressed as xylose (X1; in blue; standard deviation < 0.1%), xylobiose (X2; in green; standard deviation < 0.2%) and total xylosyl residues (Total X; in red) released from total xylan in WUS (%).

To study whether the accessibility of xylan for enzymes in the compost samples of the gypsum-free Phase I was indeed higher compared to control-composts due to higher pH, both wP-13- and wA-13-samples were subjected to a pure endo-xylanase (*Aa*GH10) hydrolysis [15]. Xylose, xylobiose and total soluble xylooligomers were quantified after hydrolysis and presented as percentage of total xylosyl residues present in the corresponding WUS samples (Fig 3).

*Aa*GH10 hydrolysis of wP0-13 or wA0-13 (Fig 3), both prepared from compost obtained at the start of Phase I, showed a similar release of xylose (0.5% ± 0.1), xylobiose (1.0% ± 0.1) and total xylooligomers (3.5% ± 0.2). For compost-WUS obtained at the end of Phase I, the percentage of xylose and xylobiose released from wP5-13 and for wA-13 (1.5% ± 0.1 and 4.2% ± 0.3, respectively) were almost similar, while the percentage total xylooligomers released for wA5-13 (13.9% ± 0.0) was higher than for wP-13 (12.1% ± 0.3). These data suggest that, indeed, a higher pH and ammonia content in Phase I as obtained in absence of gypsum led to compost in which the xylan present was more accessible for the pure endo-xylanase used. This higher accessibility for enzymes did not influence the water binding capacity of the WUS samples; no difference in water binding was analysed for all samples of P-13 or wA-13 (from 1.7 to 1.5 water bound per total carbohydrate (w/w %), standard deviation < 0.1; S2 Table).

### Composition and content of composts

The chemical compositions of all Phase I-compost samples obtained in presence or in absence of gypsum are shown in Table 1.

**Table 1:** Composition of Phase I-compost samples (2013 and 2015) of wheat straw compost obtained in presence (P) and in absence (A) of gypsum. Standard deviations for total carbohydrates content (% w/w) < 3.0 and for lignin < 2.0.

	PI-0^a^				PI-1^a^		PI-2^a^		PI-3^a^		PI-4^a^		PI-5^a^			
	P^b^		A^b^		P^b^	A^b^	P^b^	A^b^	P^b^	A^b^	P^b^	A^b^	P^b^		A^b^	
	P0-13^c^	P0-15^c^	A0-13^c^	A0-15^c^	P1-13^c^	A1-13^c^	P2-13^c^	A2-13^c^	P3-13^c^	A3-13^c^	P4-13^c^	A4-13^c^	P5-13^c^	P5-15^c^	A5-13^c^	A5-15^c^
**Content (% w/w) dm**^d^																
Lignin (Klason)^e^^,^^f^	20	22	17	23	19	19	20	20	19	18	21	20	19	21	19	18
Total carbohydrates	38	46	39	48	40	40	40	39	38	38	39	37	40	45	39	46
Ash	30	22	26	19	29	26	29	27	28	26	29	27	31	25	27	26
Total nitrogen	2	2	2	2	1	1	2	2	2	2	1	2	2	2	2	2
Protein^g^	10	8	10	8	9	9	10	9	10	10	9	10	10	7	10	7
**Carbohydrate composition (mol %)**																
Arabinosyl	5	5	5	5	5	5	5	5	6	5	6	5	6	5	5	6
Xylosyl	34	35	34	36	34	33	34	33	34	33	33	34	33	34	33	34
Mannosyl	1	1	1	1	1	1	1	1	1	1	1	1	1	1	1	1
Galactosyl	2	1	1	1	2	1	2	1	2	1	2	1	2	1	2	2
Rhamnosyl	0	0	0	0	0	1	0	1	1	1	1	1	1	0	1	1
Glucosyl	57	53	54	52	56	55	56	55	56	55	57	54	57	54	55	52
Uronic acid	3	4	4	5	3	4	3	4	3	4	3	3	3	4	3	5
**Degree of Substitution (DS)**																
DS Ara^h^	15	14	14	13	16	15	16	16	17	16	17	16	18	16	17	17
DS GlcA^h^	10	12	11	13	10	11	10	11	10	11	10	10	10	13	9	13

^a^PI-0: Phase I initial mixture, PI-1, PI-2, PI-3, PI-4, PI-5 is phase I compost after 1, 2, 3, 4 and 5 days of composting time.

^b^P: Presence of gypsum within the composting process, A: Absence of gypsum within the composting process.

^c^13: Wheat straw based compost from 2013, 15: wheat straw based compost from 2015.

^d^Weight percentage is based on dry matter of composting samples and corrected for the amount of gypsum added.

^e^Corrected for ash.

^f^Calculated values as 100 minus total carbohydrates, ash, total nitrogen and protein for the "2015" samples.

^g^Nitrogen to protein conversion factor of 6.25 was used; protein content does not include N-containing salts.

^h^DS Ara = mol Ara/100 mol Xyl; DS GlcA = mol GlcA / 100 mol Xyl; abbreviations: Ara, arabinosyl; xyl, xylosyl; GlcA, glucuronic acid.

The total carbohydrate contents of the compost obtained at the start of Phase I, were 38 and 39% (w/w), for P0-13 and A0-13 respectively, and 46 and 49% (w/w) for P0-15 and A0-15. Previously, Jurak et al. (2014) [5] reported 34% (w/w) carbohydrates to be present in compost WUS at the start of Phase I, while ash and lignin were reported to be both 25% w/w. The observed differences of the initial compost compositions are, most likely, the result of year-to-year variations in ingredient or raw material composition [20,21].

The total carbohydrate content was measured through Phase I for compost obtained from both P-13 and A-13, showing no change from day 0 to day 5 (from 38 to 40% w/w for P-13 and from 39 to 39% w/w for A-13). Likewise, no changes were found for P-15 and A-15 from day 0 to day 5 (from 46 to 45% w/w for P-15 and from 48 to 46% w/w for A-15). Previously, Jurak et al. (2014) [5] and Iiyama et al. (1994) [26] reported 38 and 40% of total carbohydrates in compost obtained at the end of Phase I, which is similar to our data.

The carbohydrate compositions (mol %) were similar for all samples obtained during Phase I. For all samples, glucosyl (52–57 mol %) and xylosyl (33–36 mol %) residues were the main carbohydrate constituents of the compost originating from the wheat straw cellulose and xylan. Additionally, arabinosyl (5–6 mol %) and glucuronyl residues (3–5 mol %) were analysed in all composts, most likely mainly present as xylan substituents as expected based on previously reported xylan structures [5]. The degree of substitution of xylosyl residues were found to be similar in our study compared to Jurak et al. (2014) [5] for P0-13 and P1-13.

In short, for all samples obtained through the composting Phase I, no major difference in their chemical composition was found regardless presence or absence of gypsum. Contents of carbohydrates, lignin, ash, total nitrogen and proteins were not affected during Phase I, matching with Jurak et al,. (2015) [12] data. Previously, Li and Kim (2011) [25] also showed that low ammonia concentrations within pretreatments of corn stover has no effect on the composition of the material obtained. Those results are in accordance to our data.

### Dry matter and carbohydrate recoveries and composition of WUS

The dry matter recovery and composition of WUS was analysed for all compost samples collected during Phase I. The yield and composition of all WUS is presented in Table 2. Recoveries of dry matter after water extraction were similar for composts obtained at the start and at the end of Phase I (74–82% for w0 (P-13, P-15, A-13 and A-15) and 73–81% for w5 (P-13, P-15, A-13 and A-15). Previously, recovery of dry matter was reported to be 82% [5] for end of Phase I being similar to our data.

**Table 2:** Recoveries and chemical composition of water unsoluble solids (WUS) of wheat straw based phase I composts that have been supplemented (P) or not (A) with gypsum, based on dry matter (DM). Standard deviation for total carbohydrates content (% w/w) dm < 3.0.

wPI-0^a^				wP1-1^a^		wPI-2^a^		wPI-3^a^		wPI-4^a^		wPI-5^a^			
wP^b^		wA^b^		wP^b^	wA^b^	wP^b^	wA^b^	wP^b^	wA^b^	wP^b^	wA^b^	wP^b^		wA^b^	
wP0-13^c^	wP0-15^c^	wA0-13^c^	wA0-15^c^	wP1-13^c^	wA1-13^c^	wP2-13^c^	wA2-13^c^	wP3-13^c^	wA3-13^c^	wP4-13^c^	wA4-13^c^	wP5-13^c^	wP5-15^c^	wA5-13^c^	wA5-15^c^
**WUS recoveries (%)**															
Total DM															
82	74	82	76	81	81	80	81	81	80	80	80	81	74	80	73
Total carbohydrates															
95	93	95	91	96	102	95	107	106	102	106	109	104	96	100	90
Glucan															
94	91	94	89	95	99	95	107	103	102	101	111	101	94	98	92
Xylan															
97	97	95	97	93	107	97	110	110	107	110	107	107	102	107	93
**Content (% w/w) DM**^d^															
Total carbohydrates															
45	58	44	58	47	51	47	51	50	49	51	51	51	58	49	57
Glucan															
26	32	26	32	28	29	28	30	29	29	30	30	30	33	29	33
Xylan															
13	18	13	20	14	15	14	15	15	15	15	15	15	18	15	18
Arabinan															
2.3	2.8	2.2	3.2	2.4	2.6	2.4	2.5	2.7	2.6	2.6	2.7	2.6	2.7	2.6	3.3
**Carbohydrate composition (mol %)**															
Arabinosyl															
5	5	5	5	5	5	5	5	5	5	5	5	5	5	5	6
Xylosyl															
34	36	34	39	33	35	35	34	35	35	34	33	34	36	35	36
Mannosyl															
1	1	1	1	1	1	1	1	1	1	1	1	1	1	1	1
Galactosyl															
1	2	1	1	1	1	1	1	1	1	1	1	1	2	1	1
Rhamnosyl															
0	0	0	0	0	0	0	0	1	0	0	0	0	0	0	0
Glucosyl															
55	52	54	51	56	54	56	55	54	54	54	55	55	53	54	53
Uronic acid															
4	4	4	3	4	4	4	4	3	3	4	4	4	3	3	3
**Degree of substitution (DS)**															
DS Ara^e^															
15	13	15	14	15	15	15	14	16	15	15	16	15	13	15	16
DS GlcA^e^															
13	10	13	8	12	11	11	11	9	10	12	11	11	9	9	9

^a^wPI-0: water unsoluble solids Phase I initial mixture, wPI-1, wPI-2, wPI-3, wPI-4, wPI-5 is water unsoluble solids Phase I compost after 1, 2, 3, 4 and 5 days of composting time.

^b^P: Presence of gypsum within the composting process, A: Absence of gypsum within the composting process.

^c^13: Wheat straw based compost from 2013, 15: wheat straw based compost from 2015.

^d^Weight percentage is based on dry matter of composting phases and corrected for the amount of gypsum added.

^e^ DS Ara = mol Ara/100 mol Xyl; DS GlcA = mol GlcA / 100 mol Xyl; abbreviations: Ara, arabinosyl; xyl, xylosyl; GlcA, glucuronic acid.

Cellulose, analysed as glucan, and xylan remained completely in the WUS for all samples analysed, which indicated that none of the carbohydrates became water soluble during Phase I composting (Table 2). Like for the composts, the main constituents of WUS-carbohydrates were glucosyl (51–56 mol %) and xylosyl (33–39 mol %) residues and the main xylan substituents were arabinosyl (DS 14–16) and glucuronyl (DS 9–13; UA) residues. The type and amount of xylan substituents, reflected in the degree of substitution (DS) (Table 2), were neither affected by presence or absence of gypsum nor by the duration of Phase I.

To conclude, the recoveries of insoluble matter and carbohydrates, compositions of WUS and type and amount of xylan substituents was similar for composts obtained at the start and end of both the gypsum-free Phase I and the Phase I control process in presence of gypsum. Hence, the higher enzyme degradability observed for the compost-WUS-samples obtained at the end of Phase I and of the gypsum-free Phase I is not related to the analysed carbohydrate content and composition of the WUS-samples.

### Lignin analysis of WUS compost by pyrolysis GC/MS

Compost samples in presence and in absence did not show differences in term of overall compositions and contents. A detailed lignin fingerprint obtained using pyrolysis GC/MS was used to investigate whether differences within the lignin component from the two experiments during Phase I were obtained. Pyrolysis GC/MS was performed in triplicate on all WUS obtained during Phase I from compost in presence and in absence of gypsum (wP-13 and wA-13) from day 0 to day 5. The identities and relative abundances (average of triplicates) of the compounds formed upon pyrolysis are shown in Table 3. The pyrolysis of untreated wheat straw released similar compounds (derived from carbohydrates and lignin) as compost-WUS-samples, although in different proportions [27], and similarly as previously reported by Jurak et al. (2015) [12]. Interestingly, the sum of the relative abundances of the compounds derived from lignin decreased in WUS obtained from day 0 to day 5; 42.8 to 33.9% for w0P-13 to w5P-13 and 46.9 to 33.1% for w0A-13 to w5A-13. The main decrease was observed from day 0 to day 1 from 42.8 to 35.6% and from 46.9 to 34.1% (for wP-13 and wA-13, respectively) and then remained stable around the value of day 1. In a previous study, the composting process was evaluated for its mass balance showing minor losses of lignin content during composting Phase I [12]. The later research published a decrease in the total relative pyrogram area from 86% ± 7 for Phase I-day 0 to 70% ± 7 for end of Phase I. Those results are in accordance with the decrease of the relative abundance of lignin within our samples.

**Table 3:** Identity and relative abundance (average of triplicates) of the compounds obtained upon pyrolysis GC/MS of WUS wheat straw-based compost in presence (wP-13) and in absence (wA-13) of gypsum after 0, 1, 2, 3, 4 and 5 days of Phase I.

Based on Pyrolysis GC-MS	wP0-13	wP1-13	wP2-13	wP3-13	wP4-13	wP5-13	wA0-13	wA1-13	wA2-13	wA3-13	wA4-13	wA5-13
% Carbo-hydrates^a^	57.2 ±2.3	64.4 ±2.1	64.5	62.9	67.7	66.1	53.1	65.9	65.3	65.1 ±3.5	66.5 ±2.3	67.0 ±3.1
% Lignin	42.8	35.6	35.5	37.1	32.3	33.9	46.9	34.1	34.7	34.9	33.5	33.0
Ratio Lignin / Carbohydrate	0.7	0.6	0.6	0.6	0.5	0.5	0.9	0.5	0.5	0.6	0.5	0.5
SUM H^b^	33.9	30.8	31.7	29.8	30.6	31.7	28.0	32.8	34.0	31.5	30.7	30.8
SUM G^b^	47.7	49.2	49.1	48.8	48.4	47.1	48.3	50.0	46.3	48.5	48.6	48.7
SUM S^b^	18.5	20.0	19.2	21.4	21.0	21.2	23.7	17.2	19.7	20.0	20.7	20.5
Ratio Syringyl / Guaiacyl	0.39	0.41	0.39	0.44	0.43	0.45	0.49	0.34	0.43	0.41	0.42	0.42
Ratio Syringyl / Guaiacyl _except vinylguaiacol_^c^	0.75	0.76	0.78	0.79	0.81	0.84	0.91	0.68	0.80	0.75	0.77	0.74
% Cα-unsubstituted lignin	37.9	33.7	39.4	37.0	44.3	46.2	43.2	40.1	42.2	43.4	44.2	44.6
% Cα-methylated lignin	7.7	7.5	8.2	7.9	7.8	8.7	6.3	7.3	7.8	8.1	7.7	8.2
% Cα-vinyl lignin	51.4	51.8	52.2	48.2	49.8	49.8	48.4	53.5	51.5	49.2	49.5	47.9
% Cα-oxidized lignin	7.3	8.7	8.5	9.8	9.1	8.9	7.2	7.9	9.1	9.3	9.4	9.8
% Cα-oxidized G-units	4.5	5.3	5.2	5.7	5.4	5.3	4.1	4.9	5.5	5.8	5.4	5.9
% Cα-oxidized S-units	2.9	3.4	3.3	4.1	3.7	3.6	3.1	2.9	3.6	3.5	3.9	3.9

^a^ Standard deviation of the % of carbohydrates were < 1.5, except shown differently.

^b^ Standard deviations of the sum of H, G and S compounds were < 1.5, except shown differently.

^c^ All G and S derived peaks were used for the estimation of S:G ratio except 4-vinylguaiacol which also can arise from ferulates.

Lignin derived compound can be classified according the three main monolignols units: *p*-hydroxyphenyl (H), guaiacyl (G) and syringyl (S) [28,29]. Furthermore, lignin-derived H, G or S units can be classified according to their aromatic-ring substituent such as Cα-unsubstituted lignin (e.g. guaiacol), Cα-methylated lignin (e.g. 4-methylguaiacol), Cα-vinyl lignin (e.g. 4-vinylguaiacol) or Cα-oxidized lignin (e.g. vanillin) [28,29].

The composting process had different effects on lignin H, G and S units. The sum of H was similar after 5 days for wP5 and wA5 (31.7% and 30.8%, respectively) even if the sum of H of wP0 was 33.9% and 28.0 for wA0. The sum of G remained stable from day 0 to day 5 for both tunnels, ranging from 47.7 to 47.1% for wP and from 48.3 to 48.7% for wA. Similarly to H units, the sum of S was similar after 5 days for wP5 and wA5 (21.2% and 20.5%, respectively) even if the sum of H of wP0 was 18.5% and 23.7% for wA0. Comparing both tunnels, no major differences could be found concerning the sum of H, G and S.

Unlike the type of lignin unit (H, G and S) abundance, the functional group attached to the aromatic units were affected over the process (Table 3). The percentages representing Cα-unsubstituted lignin decreased from day 0 (37.9 and 43.2% for wP0 and wA0, respectively) to day 1 (33.7 and 40.1% for wP1 and wA1, respectively) to increase again until day 5 (46.2 and 44.6% for wP5 and wA5, respectively). Cα-methylated lignin and Cα-oxidized lignin increased from day 0 to day 5. The percentage of Cα-vinyl lignin, on the contrary, showed a minor decrease from day 0 to day 5. No major differences can be observed between wP-13 and wA-13 among lignin-substituents distribution.

Most pronounced was the decrease in 4-vinylphenol (compound 20) and 4-vinylguaiacol (compound 25), that can be derived from *p*-coumarates and ferulates [30,31], respectively, from day 0 to day 5 (from 10.6 to 8.0% and from 10.0 to 7.7% for wP-13 and wA-13, respectively) for 4-vinylphenol and for 4-vinylguaiacol (from 9.9 to 7.5% and from 10.4 to 6.9% for wP-13 and wA-13, respectively) (S3 Table). The later decrease may relate to a decrease in ester-linkages between xylan and lignin as previously described by Liu et al. (2013) [23] as a result of high ammonia concentration (>10 wt %) treatment (temperature > 100°C). The same was observed by Murciano et al. (2016) [32] for NaOH treated sugar cane bagasse showing a decrease of ester linked *p*-coumarates and ferulates, which positively affect enzymatic hydrolysis. Our data confirm our hypothesis that higher pH increased enzymatic hydrolysis, expectedly by opening the xylan-lignin structure by breaking ester-linked bounds.

## Conclusions

In this study, a composting Phase I process was conducted in absence of gypsum and compared to the control process in presence of gypsum. Optimal compost properties were reached after 2 and 4 days in absence of gypsum compared to 3 and 5 days in the control. Glucan and xylan enzymatic degradation yields (34 and 23%, respectively) were reached 1 day earlier in absence of gypsum. These results show the potential of a faster “gypsum-free” alternative for Phase I. Additionally, composting Phase I was confirmed to be crucial leading to a decrease of 4-vinylphenol and 4-vinylguaiacol that can be associated with *p*-coumarates and ferulates linking lignin and xylan. Thus, authors recommend to introduce gypsum within the process by the end of Phase I accordingly to Phase II condition requirements.

## Supporting information

S1 Table Moisture content (w/w % dry matter based) measured during the first 5-days of the composting process (Phase I) from a wheat straw-manure based mixture. Std < 0.1.
(PDF)

S2 Table Water binding capacity of WUS compost samples expressed as ratio of water bound per total carbohydrates (std < 0.1), per total glucans (std < 0.1) and per total xylan (std < 0.2). (PDF)

S3 Table Identities and relative abundance (average of triplicates) of the compounds obtained and detected upon pyrolysis GC/MS of WUS wheat straw-based compost in presence (wP-13) and in absence (wA-13) of gypsum after 0, 1, 2, 3, 4 and 5 days of Phase I. (PDF)
