# Supplementary material for: Potential of a gypsum-free composting process of wheat straw for mushroom production
Source: PLoS One. 2017 Oct 5;12(10):e0185901. doi: 10.1371/journal.pone.0185901 (PMC5628895; doi:10.1371/journal.pone.0185901)
Supplement: S1 Table — Std < 0.1. (PDF) [file pone.0185901.s001.pdf]

S1 Table: Moisture content (w/w % dry matter based) measured during the first 5-days of the composting process (Phase I) from a wheat straw-manure based mixture. Std < 0.1

w/w %	PI-0		PI-1		PI-2		PI-3		PI-4		PI-5	
	P0-13	A0-13	P1-13	A1-13	P2-13	A2-13	P3-13	A3-13	P4-13	A4-13	P5-13	A5-13
Moisture per dry matter	74.09	75.18	73.53	74.30	72.20	73.35	73.63	74.07	73.18	73.60	72.76	73.65
