# Supplementary material for: Potential of a gypsum-free composting process of wheat straw for mushroom production
Source: PLoS One. 2017 Oct 5;12(10):e0185901. doi: 10.1371/journal.pone.0185901 (PMC5628895; doi:10.1371/journal.pone.0185901)
Supplement: S2 Table — (PDF) [file pone.0185901.s002.pdf]

S2 Table: Water binding capacity of WUS compost samples expressed as ratio of water bound per total carbohydrates (std < 0.1), per total glucans (std < 0.1) and per total xylan (std < 0.2).

	PI-0		PI-1		PI-2		PI-3		PI-4		PI-5	
	wP0-13	wA0-13	wP1-13	wA1-13	wP2-13	wA2-13	wP3-13	wA3-13	wP4-13	wA4-13	wP5-13	wA5-13
Water bound per total carbohydrates	1.6	1.7	1.7	1.6	1.7	1.5	1.6	1.6	1.5	1.5	1.6	1.5
Water bound per total glucan	2.8	2.9	2.8	2.8	2.9	2.6	2.7	2.7	2.7	2.5	2.7	2.7
Water bound per total xylan	5.6	5.6	5.7	5.2	5.7	5.1	5.2	5.2	5.2	5.1	5.4	5.0
