# Supplementary material for: Potential of a gypsum-free composting process of wheat straw for mushroom production
Source: PLoS One. 2017 Oct 5;12(10):e0185901. doi: 10.1371/journal.pone.0185901 (PMC5628895; doi:10.1371/journal.pone.0185901)
Supplement: S3 Table — (PDF) [file pone.0185901.s003.pdf]

S3 Table: Identities and relative abundance (average of triplicates) of the compounds obtained and detected upon pyrolysis GC/MS of WUS wheat straw-based compost in presence (wP-13) and in absence (wA-13) of gypsum after 0, 1, 2, 3, 4 and 5 days of Phase I.

Label	compound	Origin[a]	wP0-13	wP1-13	wP2-13	wP3-13	wP4-13	wP5-13	wA0-13	wA1-13	wA2-13	wA3-13	wA4-13	wA5-13
1	2-methylfuran	C	0.4	0.2	0.2	0.2	0.2	0.2	0.3	0.0	0.4	0.1	0.2	0.1
2	Furfural	C	8.7	7.7	8.6	8.0	8.8	8.5	8.6	7.9	6.9	8.1	8.3	8.2
3	2-furanmethanol	C	1.2	0.7	0.9	0.8	0.8	1.0	1.7	0.8	0.7	0.8	0.8	0.9
4	2-acetylfuran	C	0.3	0.2	0.2	0.2	0.2	0.2	0.4	0.1	0.2	0.2	0.2	0.2
5	2,3-dihydro-5-methylfuran	C	7.5	4.2	6.1	5.8	5.5	6.5	11.6	5.4	5.6	6.2	5.3	5.9
6	2(5H)-furanone	C	5.1	2.3	3.2	2.9	3.0	3.1	7.1	3.1	3.6	3.1	3.5	3.4
7	5-ethyl-2-furaldehyde	C	0.3	0.2	0.3	0.3	0.3	0.3	0.2	0.2	0.3	0.3	0.3	0.3
8	5-(hydroxymethyl)dihydro-2(3H)-furanone	C	1.2	0.6	0.8	0.7	0.6	0.8	2.0	0.7	1.1	0.8	0.8	0.9
9	1,4-anhydroarabinofuranose	C	1.6	1.7	2.1	2.1	2.1	1.9	1.2	1.9	2.1	2.2	2.2	2.5
10	5-(hydroxymethyl)-2-Furancarboxaldehyde	C	1.7	2.5	2.3	2.6	2.3	2.4	1.1	2.2	2.4	2.3	2.5	2.6
11	1,6-anhydro-β-D-glucopyranose (levoglucosan)	C	8.4	15.3	14.3	13.9	15.2	15.3	5.0	14.8	16.8	13.7	13.9	14.6
12	2,4-dihydropyran-3-one	C	0.4	0.4	0.4	0.3	0.4	0.4	0.5	0.5	0.6	0.4	0.4	0.4
13	5-hydroxy-2-tetrahydrofuraldehyde-3-one	C	0.9	1.5	1.4	1.5	1.4	1.5	0.4	1.4	1.7	1.6	1.6	1.5
14	1,4-anhydroxylofuranose	C	1.6	2.9	2.6	2.5	2.9	2.6	1.2	2.6	2.5	2.4	2.6	2.8
15	4-hydroxy-5,6-dihydro-2H-pyran-2-one	C	17.9	24.1	21.1	21.2	24.0	21.2	11.8	24.2	20.3	22.7	24.0	22.7
16	Phenol	H	2.2	1.1	1.6	1.5	1.4	1.6	2.1	1.5	1.7	1.7	1.5	1.4
17	2-methylphenol (o-cresol)	H	0.0	0.0	0.0	0.0	0.0	0.0	0.0	0.0	0.0	0.0	0.0	0.0
18	4-methylphenol (p-cresol)	H	1.3	0.6	0.9	0.8	0.7	0.9	0.8	0.7	0.9	0.8	0.7	0.8
19	4-ethylphenol	H	0.3	0.2	0.2	0.2	0.2	0.2	0.2	0.2	0.2	0.2	0.2	0.2
20	4-vinylphenol	H / PCA	10.6	9.0	8.5	8.5	7.6	8.0	10.0	8.7	9.0	8.3	7.9	7.7

21	Hydroquinone	H	0.0	0.0	0.0	0.0	0.0	0.0	0.0	0.0	0.0	0.0	0.0	0.0
22	Guaiacol	G	3.1	1.9	2.2	2.3	2.1	2.4	4.4	2.1	2.3	2.4	2.3	2.2
23	4-methylguaiacol	G	1.3	1.3	1.3	1.4	1.2	1.3	1.4	1.2	1.2	1.4	1.3	1.3
24	4-ethylguaiacol	G	0.4	0.4	0.3	0.3	0.3	0.3	0.4	0.3	0.4	0.4	0.4	0.4
25	4-vinylguaiacol	G / FA	9.9	8.1	8.7	8.0	7.2	7.5	10.4	8.4	7.6	7.6	7.4	6.9
26	Eugenol	G	0.2	0.2	0.2	0.2	0.2	0.2	0.3	0.2	0.2	0.3	0.2	0.2
27	Vanillin	G	1.1	1.1	1.0	1.2	0.9	0.9	0.9	0.9	1.0	1.1	1.0	1.0
28	Acetovanillone	G	0.5	0.5	0.5	0.6	0.5	0.6	0.5	0.5	0.6	0.6	0.5	0.6
29	Guaiacylacetone	G	0.3	0.3	0.3	0.3	0.2	0.3	0.5	0.3	0.4	0.3	0.3	0.3
30	*cis*-isoeugenol	G	0.2	0.1	0.1	0.1	0.1	0.1	0.2	0.1	0.1	0.2	0.1	0.1
31	guaiacyl vinyl ketone	G	0.1	0.1	0.1	0.1	0.1	0.1	0.1	0.1	0.2	0.1	0.1	0.1
32	*cis*-coniferyl-alcohol	G	0.2	0.2	0.2	0.2	0.2	0.2	0.2	0.2	0.2	0.2	0.2	0.2
33	*trans*-coniferyl-alcohol	G	2.7	2.8	2.0	2.8	2.1	1.8	3.1	2.3	1.6	2.0	2.2	2.2
34	*trans*-coniferaldehyde	G	0.3	0.4	0.4	0.4	0.4	0.3	0.3	0.4	0.4	0.4	0.4	0.4
35	Syringol	S	1.6	1.2	1.2	1.3	1.2	1.4	3.1	1.1	1.2	1.2	1.2	1.2
36	4-methylsyringol	S	0.7	0.7	0.7	0.7	0.6	0.7	0.8	0.5	0.6	0.7	0.6	0.6
37	4-ethylsyringol	S	0.2	0.1	0.2	0.2	0.1	0.2	0.3	0.1	0.1	0.1	0.1	0.2
38	4-vinylsyringol	S	1.5	1.3	1.3	1.5	1.3	1.4	2.3	1.1	1.3	1.3	1.3	1.2
39	4-allyl-2,6-dimethoxyphenol	S	0.2	0.2	0.2	0.2	0.2	0.2	0.2	0.1	0.2	0.2	0.2	0.2
40	Syringaldehyde	S	0.4	0.5	0.4	0.6	0.5	0.5	0.5	0.4	0.5	0.5	0.6	0.5
41	Acetosyringone	S	0.5	0.5	0.5	0.6	0.5	0.5	0.6	0.4	0.5	0.5	0.5	0.5
42	Syringylacetone	S	0.3	0.2	0.2	0.3	0.2	0.2	0.4	0.2	0.2	0.2	0.2	0.2
43	*cis*-2,6-dimethoxy-4-propenylphenol	S	0.1	0.1	0.1	0.1	0.1	0.1	0.2	0.1	0.1	0.1	0.1	0.1
44	1-(3,5-dimethoxy-4-hydroxyphenyl)propyne	S	0.1	0.2	0.1	0.1	0.2	0.2	0.1	0.1	0.1	0.1	0.2	0.1
45	*trans*-2,6-dimethoxy-4-propenylphenol	S	1.0	0.9	0.9	1.0	0.8	1.0	1.2	0.7	0.9	1.0	0.8	0.8
46	Homosyringaldehyde	S	0.2	0.2	0.2	0.1	0.3	0.1	0.2	0.2	0.3	0.2	0.2	0.2
47	*cis*-sinapyl-alcohol	S	0.1	0.1	0.1	0.1	0.1	0.1	0.1	0.1	0.0	0.1	0.1	0.1
48	*trans*-sinapyl-alcohol	S	0.9	0.8	0.5	0.9	0.6	0.5	1.1	0.5	0.5	0.5	0.6	0.6
49	*trans*-sinapaldehyde	S	0.2	0.2	0.2	0.2	0.2	0.2	0.2	0.2	0.2	0.2	0.2	0.2
Label	compound	Origin[a]	wP0-13	wP1-13	wP2-13	wP3-13	wP4-13	wP5-13	wA0-13	wA1-13	wA2-13	wA3-13	wA4-13	wA5-13

[a] C, carbohydrate-derived compound; H, *p*-hydroxycinnamyl lignin-derived compounds; G, guaiacyl lignin-derived compounds; S, syringyl lignin-derived compounds; PCA, p-coumarates; FA, ferulates.
